# Fermentation of *Pediococcus pentosaceus* JC30 Improves Phytochemical, Flavor Characteristics and Antioxidant Activity of Mulberry Leaves

**DOI:** 10.3390/molecules30081703

**Published:** 2025-04-10

**Authors:** Caiyan Meng, Jiawen Xie, Jiaqi Chen, Jiajia Xuan, Zhuoying Zeng, Minghua Lai, Xuerui Kang, Jiayun Li, Guanhui Liu, Jie Tu, Hongxun Tao

**Affiliations:** 1 College of Biotechnology, Jiangsu University of Science and Technology, Zhenjiang 212003, China; mengcaiyan00@163.com (C.M.); xiejiawen0819@163.com (J.X.); 222211801202@stu.just.edu.cn (J.C.); jiajiaxuan99@163.com (J.X.); 222211801201@stu.just.edu.cn (Z.Z.); 222211802119@stu.just.edu.cn (M.L.); 222211801107@stu.just.edu.cn (X.K.); 222211801108@stu.just.edu.cn (J.L.); 2 School of Grain Science and Technology, Jiangsu University of Science and Technology, Zhenjiang 212008, China; liuguanhui@just.edu.cn; 3 School of Food and Biological Engineering, Jiangsu University, Zhenjiang 212013, China

**Keywords:** *Pediococcus pentosaceus* JC30, mulberry leaves, volatile compounds, bioactive compounds, antioxidant activity

## Abstract

Mulberry leaves contain polysaccharides, phenols, alkaloids, and other active ingredients which have medicinal and edible value. In this study, fermented mulberry leaf powder was prepared by solid-state fermentation using *Pediococcus pentosaceus* JC30. The effects of the fermentation on the phytochemical, flavor characteristics, phenolics, and antioxidant activity of mulberry leaves were studied. The results showed that the content of γ-aminobutyric acid in fermented mulberry leaf powder (FMLP) increased by 6.73-fold and the content of phytic acid decreased by 11.16%. Ultra-high-performance liquid chromatography with quadrupole time-of-flight mass spectrometry (UPLC-Q-TOF-MS) analysis showed that the fermentation of *Pediococcus pentosaceus* JC30 altered the phenolic composition of mulberry leaves, increasing the total free phenolic content by 88.43%. In particular, the contents of free phenols such as leucocyanidin, myricetin, and quercetin increased significantly and were positively correlated with antioxidant capacity. The fermentation of *Pediococcus pentosaceus* JC30 significantly enhanced the scavenging ability of DPPH free radicals, hydroxyl radicals, and the total reducing ability of mulberry leaves. Gas chromatography ion mobility spectrometry (GC-IMS) analysis showed that FMLP has an intense fruity and floral aroma, while having less grassy and earthy odor. The fermentation improved the phytochemical, flavor, and nutritional value of mulberry leaves, which provides more possibilities for the development of mulberry leaf products outside the sericulture industry.

## 1. Introduction

Mulberry leaves come from the mulberry tree (*Morus alba* L.), which is a deciduous tree or shrub belonging to the mulberry family. In China, mulberry leaves have always been the main feed for silkworms and have supported the silk industry. Nowadays, mulberry leaves are recognized as a natural product widely used for food, medicine, livestock and aquatic feed, as well as cosmetics, with high economic value [1,2]. Mulberry leaves contain various active ingredients, including polysaccharides, phenolics, flavonoids, free amino acids, γ-aminobutyric acid (GABA), 1-deoxynojirimycin (DNJ), etc. [3]. Therefore, they have physiological activities such as antioxidant, cardioprotective, anticancer, antibacterial, and anti-inflammatory effects [2]. However, for the development of food or feed products, mulberry leaves have many unfavorable limiting factors. Mature mulberry leaves contain high levels of crude fiber, anti-nutritional factors (such as phytic acid and tannins), and a heavy, grassy flavor [4].

Microbial fermentation is an effective processing method that can increase the nutritional value and improve the flavor of raw materials. Chuah et al. reported that *Lactobacillus plantarum* TAR4, baker’s yeast, red yeast, tempeh, and tapai starter were used to ferment mulberry leaves and fruits, respectively. The results showed that the DPPH scavenging capacity and α-amylase inhibitory activity of mulberry leaves and mulberry fruits were all significantly increased by the above start cultures’ fermentation (*p* < 0.05). Among them, *Lactobacillus plantarum* TAR4 fermentation increased the DPPH scavenging ability of mulberry juice by about 45% [5]. Therefore, lactic acid bacteria (LAB) fermentation is a feasible way to improve the antioxidant capacity of mulberry leaves or fruits. Guan et al. prepared fermented mulberry juice with *Lactobacillus plantarum* BXM2 isolated from naturally fermented honey passion fruit beverage to improve the flavor profile and increase the contents of various bioactive substances. The results showed that 64 amino acids and derivatives, 49 flavonoids, 12 organic acids, and 56 lipid metabolites were increased by fermentation, and the fermented mulberry juice had a persistent and strong lipid, floral, fruity, fermented, and sour aroma [6]. Wang et al. found that *Lactobacillus casei* reduced the pH value of animal feeds, thereby inhibiting the growth of undesirable microorganisms [7]. In summary, microbial fermentation has great potential to enhance the nutritional value, flavor, and shelf life of mulberry leaf products. In addition, it has been found that fermentation can bring more health benefits. For example, using *Lactobacillus plantarum* fermentation, Tang et al. enhanced mulberry residue’s α-glucosidase inhibitory activity, achieving 93.41% inhibition at 0.05 mg/mL in vitro. The fermentation also reduced the glucose content and reactive oxygen species (ROS) levels in *Caenorhabditis elegans* [8]. Xiao et al. reported that the fermentation supernatant rich in GABA improved cellular oxidative stress by reducing ROS and malondialdehyde levels [9]. Chen et al. found that fermentation by *Lactobacillus plantarum* and *Lactobacillus acidophilus* increased the content of gallic acid, protocatechuic acid, caffeic acid, and other active components in strawberry juice, thereby enhancing antioxidant capacity [10].

*Pediococcus pentosaceus* (*P. pentosaceus*) is a promising LAB with anti-inflammatory, anticancer, antioxidant, detoxification, and lipid-lowering abilities [11]. Kuppusamy et al. reported that *P. pentosaceus* S22 significantly improved the antioxidant capacity of silage, which indicated that *P. pentosaceus* S22 could provide a new approach for the development of legume silage inoculants [12]. *P. pentosaceus* JS91 and *P. pentosaceus* JS96 were screened from *Jiangshui* by Jin et al. and were used to ferment pear juice. The results showed that the fermented pear juice had a lower pH and high titratable acidity. Additionally, the fermentation of *P. pentosaceus* JS91 and JS96 significantly increased the scavenging capacity of superoxide anion radicals and the total antioxidant capacity of pear juice [13]. In our previous research, *P. pentosaceus* JC30 was screened for its GABA-producing capacity [14], which has potential to improve the nutritional value of the fermented products.

To promote the consumption of mulberry leaves, *P. pentosaceus* JC30 was used to prepare fermented mulberry leaf powder by solid-state fermentation (SSF) for further food or feed processing. The changes in the physicochemical composition, flavor, and antioxidant activity of mulberry leaf powder before and after fermentation were systematically studied in this study. The results will provide an experimental basis for the effects of *P. pentosaceus* JC30 on the flavor and nutritional value of mulberry leaves and broaden the way for the development and utilization of mulberry leaf resources.

## 2. Results and Discussion

### 2.1. Analysis of pH, Total Titratable Acidity, and Organic Acids

**Figure 1:**
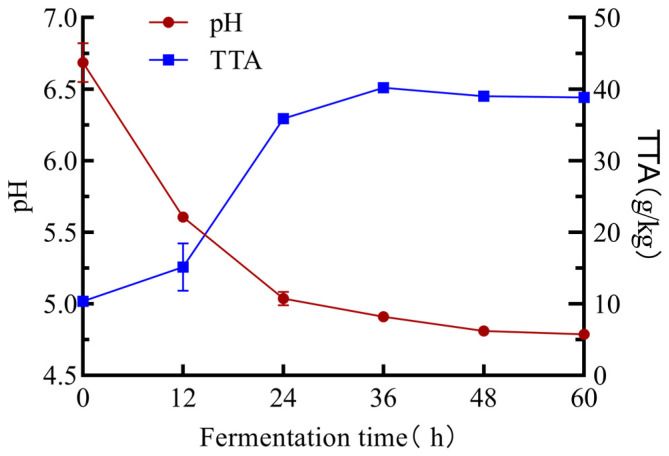
Effects of SSF on the pH and TTA of mulberry leaf powder.

The pH and total titratable acidity (TTA) of mulberry leaf powder were measured every 12 h during the fermentation of *P. pentosaceus* JC30 (Figure 1). With increasing fermentation time, the pH first decreased and then remained stable, and the TTA values first increased and then remained stable. During fermentation, LAB decomposes and converts sugars and lipids into organic acids such as lactic acid, acetic acid, and fatty acids. This process leads to a decrease in the pH and an increase in the TTA [15].

The organic acids in unfermented mulberry leaf powder (MLP) and fermented mulberry leaf powder (FMLP) were identified by comparing their retention times with those of standards, and they were quantified by using their calibration curves and by evaluating the ultraviolet (UV) absorbance spectrum. Organic acid profile analysis (Table 1) revealed that lactic acid was the dominant organic acid in FMLP, which ranged from 0 mg/g in MLP to 15.792 mg/g in FMLP. After fermentation by *P. pentosaceus* JC 30, the contents of acetic acid, citric acid, tartaric acid, and pyruvic acid also increased significantly (*p* < 0.05). Otherwise, the contents of malic acid, pyroglutamic acid, and succinic acid significantly decreased (*p* < 0.05). LAB has been reported to be able to utilize malic acid as a carbon source, and lactic acid/malic acid conversion occurred concurrently, resulting in a decrease in the malic acid content [16]. The increase in citric acid content might be attributed to accumulation through the tricarboxylic acid cycle by LAB [17].

### 2.2. Changes in Chemical Compositions Contents

**Figure 2:**
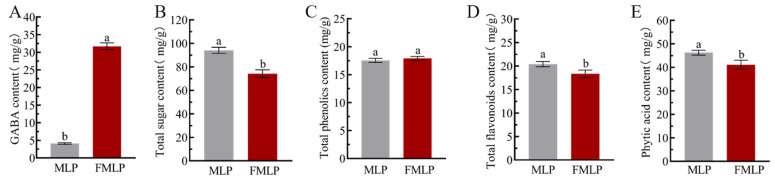
Effects of SSF on the chemical compositions of mulberry leaf powder. (**A**) GABA content. (**B**) Total sugar content. (**C**) Total phenolic content. (**D**) Total flavonoids content. (**E**) Phytic acid content. Different letters indicate a significant difference, with *p* < 0.05.

GABA is an important active component in mulberry leaves and has physiological activities, such as calming and tranquilizing, improving sleep, and anti-anxiety effects [18]. In our previous study, *P. pentosaceus* JC30 was isolated as a GABA-producing strain with acid, heat, and bile salt resistance [14]. As shown in Figure 2A, the GABA content in the FMLP group was 31.68 ± 0.97 mg/g. After 48 h of fermentation by *P. pentosaceus* JC30, the GABA content in the FMLP increased by 6.73-fold. Similarly, Zhong et al. used *Lactobacillus pentosus* SS6 screened from fermented mulberry fruit to ferment mulberry leaf powder and found that the GABA content in the fermented mulberry leaf powder increased by 4.17-fold [19]. That is to say, screening potential fermentation strains can effectively increase the content of GABA in mulberry leaf powder.

As shown in Figure 2B, the total sugar content in mulberry leaf powder decreased from 95.14 ± 2.58 mg/g to 74.24 ± 3.26 mg/g after 48 h of fermentation. Sugar is the main carbon source for microorganism growth [5]. *P. pentosaceus* JC30 utilizes the sugars in mulberry leaf powder as a carbon source for metabolism and converts sugars into other metabolites, resulting in a lower total sugar content.

Phenols and flavonoids are highly regarded bioactive components of mulberry leaves with antioxidant and hypoglycemic effects [20]. The total phenolic content (TPC) of FMLP slightly increased but not statistically significantly (Figure 2C). This might be due to the fact that enzymes from LAB converted phenolics in the bound state to phenols in the free state, while at the same time phenolics were metabolized and modified by LAB into other affixes, glycosides, or related forms [21,22]. Different types of phenolics are converted into compounds which tend to be more biologically active than the parent compound [23]. As shown in Figure 2D, the total flavonoids content (TFC) of FMLP decreased. This might be related to the rapid proliferation of LAB, which causes faster consumption and modification of flavonoids, lowering overall flavonoid levels [24]. Studies have shown that the reduction in flavonoids during microbial fermentation does not usually lead to a reduction in biological activity [23].

Phytic acid is an anti-nutritional factor in mulberry leaves that can combine with proteins, vitamins, and other nutrients to form precipitates or complexes, which are detrimental to the absorption and utilization of nutrients [4]. As shown in Figure 2E, after fermentation, the phytic acid content decreased from 46.25 ± 1.04 mg/g to 41.09 ± 1.93 mg/g. *P. pentosaceus* JC30 fermentation effectively reduces the phytic acid content. This is because that phytase was secreted by the metabolism of *P. pentosaceus* JC30, which degrades phytic acid [25].

### 2.3. Qualitative Analysis of Free and Bound Phenolic Compounds from Mulberry Leaf Powder

A total of 68 phenolic compounds were identified by UPLC-Q-TOF-MS (the relevant participant information is shown in Supplementary Table S1). Table 2 shows the changes in 15 phenolic compounds commonly found in mulberry leaves [2]. As shown in Table 2, by *P. pentosaceus* JC30 fermentation, partially bound phenolic (BP) compounds in mulberry leaves were converted into free phenolic (FP) compounds, and the total abundance of FP increased significantly (*p* < 0.05), especially that of leucocyanidin, ellagic acid_1, myricetin, and quercetin in the free state, which increased by 117.25-, 39.80-, 27.95- and 11.70-fold, respectively. The above results may be related to the increase in antioxidant capacity of MLP. Several similar results have been reported [26,27]. It was reported that LAB produced some enzymes such as glycosidase and esterase during fermentation, which promote the hydrolysis of BPs and release of FPs, leading to increased antioxidant activity [28]. In addition, studies have reported that phenolics can be transformed into each other during microbial fermentation. For example, the increase in gallic acid and ellagic acid content after LAB fermentation may be due to the tannase activity of LAB, resulting in bioconversion of gallotanins and ellargitannins [29]. Glycosidases produced by LAB catalyzed the hydrolysis of glycosidic bonds of phenolics, leading the conversion of quercetin glycosides to quercetin [30].

After fermentation of *P. pentosaceus* JC30, the abundance of neohesperidin in the free state was significantly higher than the total abundance of the bound and free states before fermentation, which is mainly due to the bioconversion of hesperidin by LAB fermentation [31]. The increase in the abundance of neohesperidin gave the FMLP a stronger fruity flavor, especially a citrus aroma [32].

In summary, the fermentation of *P. pentosaceus* JC30 significantly changed the phenolic profile of mulberry leaf powder, many phenolic compounds were transformed from the bound state to the free state, and the total amount of FPs was significantly increased, thereby significantly improving the antioxidant capacity and flavor of mulberry leaves.

### 2.4. Effects of Solid-State Fermentation (SSF) on the Antioxidant Activities of Mulberry Leaves

**Figure 3:**
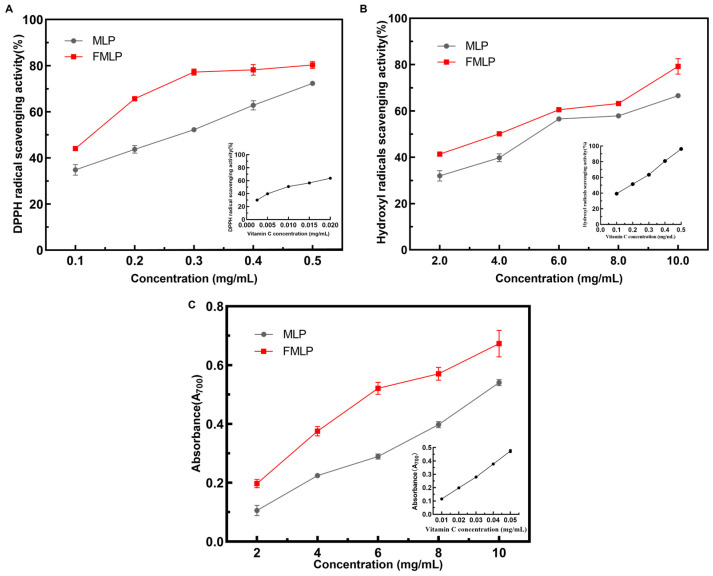
Effects of SSF on the antioxidant activities of mulberry leaves. (**A**) DPPH radical scavenging activity. (**B**) Hydroxyl radical scavenging activity (^•^OH). (**C**) Total reducing power (TRP).

The DPPH radical scavenging capacity is a commonly used indicator for evaluating the antioxidant activity of raw materials [33]. As shown in Figure 3A, *P. pentosaceus* JC30 fermentation significantly increased the DPPH radical scavenging capacity of MLP. The DPPH radical scavenging rate of MLP was 52.26 ± 0.38% at a concentration of 0.3 mg/mL, while that of FMLP was increased by 25.28%.

Hydroxyl radical (^•^OH) is one of the most active reactive oxygen species, capable of causing lipid peroxidation and biological damage, and the hydroxyl radical scavenging capacity is an important indicator for evaluating the performance of antioxidants in antioxidant assays [34]. The results in this study showed that at concentrations ranging from 2 to 4 mg/mL, the hydroxyl radical scavenging capacity of MLP ranged from 31.96 ± 2.24% to 66.56 ± 0.95%, while that of FMLP was from 41.33 ± 0.44% to 79.24 ± 3.35% (Figure 3B). Fermentation by *P. pentosaceus* JC30 thus significantly improved the ^•^OH scavenging ability of MLP. Similar results have also been reported. Tan et al. used *Lactobacillus plantarum* to ferment mulberry leaves and found the ^•^OH scavenging activity increased from 41.2 ± 1.3% to 66.8 ± 2.0% [35]. Deekshith et al. used *Lactobacillus brevis* to ferment tea leaves, and the ^•^OH scavenging capacity of the tea leaves increased from 35.4 ± 0.9% to 73.1 ± 1.5% [36]. The above research indicated that the fermentation by LAB is a promising strategy to enhance the ^•^OH scavenging capacity of plant-based materials.

Total reducing power (TRP) reflects the ability of a substance to provide electrons to reduce iron ion complexes to the ferrous form. It reflects the potential of a substance to neutralize free radicals and reactive oxygen species and is an important indicator of a substance’s antioxidant activity [37]. *P. pentosaceus* JC30 fermentation significantly increased the total reducing capacity of the sample. The results are shown in Figure 3C. The absorbance values of MLP and FMLP were 0.54 ± 0.01 and 0.67 ± 0.04, respectively, when the extract concentration was 10 mg/mL. The TRP of the FMLP was significantly stronger than that of the MLP. It was reported that LAB fermentation increased the antioxidant capacity of mulberry leaves [38]. However, the main contributors to the improvement of antioxidant capacity have not been elucidated. Therefore, the correlation between changes in the content of specific chemical substances and changes in antioxidant capacity deserves further analysis.

**Figure 4:**
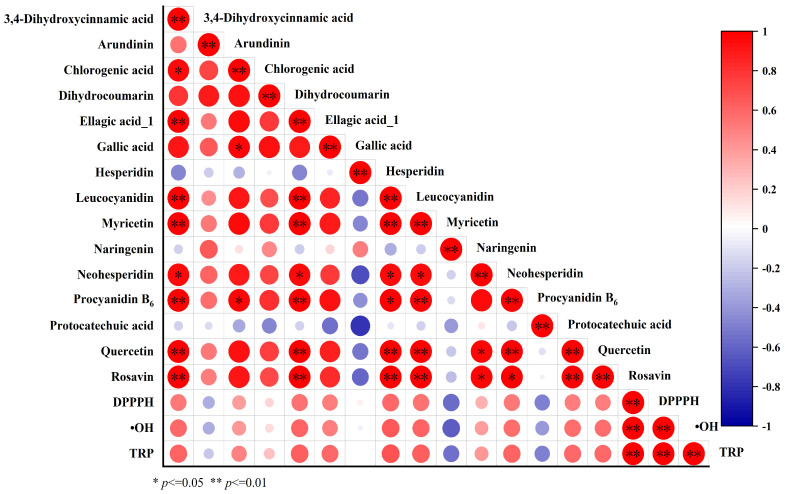
Heat map of the Pearson correction coefficient for the heat map of the phenolic profile and antioxidant activities. * Correlation is significant at *p* ≤ 0.05. ** Correlation is significant at *p* ≤ 0.01.

Phenolics are important antioxidant components. In this study, a correlation heat map was used to further analyze the relationships between phenolics and antioxidant capacity in the MLP and FMLP samples. The results revealed a positive correlation between certain phenolic compounds and antioxidant capacity. As shown in Figure 4, the contents of phenolic compounds such as leucocyanidin, myricetin, procyanidin B_6_, and quercetin were positively correlated with the DPPH radical scavenging activity. The contents of phenolic compounds such as 3,4-dihydroxycinnamic acid, ellagic acid_1, leucocyanidin, myricetin, procyanidin B_6_, and quercetin were positively correlated with the ^•^OH scavenging activity and TRP. Phenolic content is closely related to antioxidant capacity. Wang et al. reported a significant positive correlation between vanillic acid and arbutin in fermented pear juice and DPPH radical scavenging capacity [15]. In summary, the increase in phenolic content and the proportion of free phenolics after fermentation significantly improved the antioxidant capacity of the MLP, especially free phenols such as quercetin and myricetin.

### 2.5. Changes in Free Amino Acid (FAA) Content

As shown in Table 3, the total amino acid (TAA) content in the FMLP was significantly higher than that in MLP. Mulberry leaves are rich in protein, and LAB fermentation can degrade large molecules such as proteins into small molecules such as peptides and amino acids, which can be easily digested and absorbed by animals. At the same time, the LAB’s own metabolism will also provide amino acid metabolites [39]. Amino acids are classified into essential (EAAs) and non-essential amino acids (NEAAs) on the basis of their nutritional properties. After fermentation, the contents of all essential amino acids increased significantly (*p* < 0.05). Compared with MLP, the methionine (Met) content in FMLP increased from an undetected level to 18.73 ± 0.10 mg/mL, and the contents of leucine (Leu) and lysine (Lys) increased by 7.40- and 3.50-fold, respectively. It was reported that Leu is beneficial for lipid metabolism and insulin sensitivity, thus preventing and treating metabolic diseases, including type 2 diabetes and obesity [40]. Lys can improve animal growth performance and immunity and optimize the efficiency of the use of mulberry leaves as feed [41]. So, fermentation by *P. pentosaceus* JC30 effectively improved the nutritional value of mulberry leaves. Additionally, different amino acid compositions have significant effects on the flavor of food [42]. Alanine and glycine have sweetness, and increasing their contents may improve the flavor of mulberry leaves and make them more palatable. The reduction in arginine content also helped to reduce the bitterness of mulberry leaves [43]. When the percentage of EAA/TAA in food is about 40%, and the percentage of EAAs/NEAAs reaches 60%, the protein quality is excellent [44]. The average contents of EAAs/TAAs and EAAs/NEAAs in FMLP were 41.89% and 72.09%, respectively. It shows that mulberry leaves have higher nutritional value after *P. pentosaceus* JC30 fermentation and can be used as ideal protein raw materials.

It is generally believed that the higher the amino acid score (AAS) and chemical score (CS) of an essential amino acid are, the richer the essential amino acid is, and lower AAS or CS scores indicate that the nutrition of the amino acid is lower, and the amino acid with the lowest score was the limiting amino acid [45]. As shown in Table 4, the first and second limiting amino acids of MLP are Leu and phenylalanine + tyrosine, respectively. Therefore, in practical applications, these three amino acids need to be strengthened to further improve the utilization rate, and the fermentation effectively increased the content of these three essential amino acids. The AAS values of FMLP were higher than 100, indicating that its essential amino acid balance was perfect and the proportion of the amino acids was appropriate, and they were easily digested and absorbed by the body. Compared with the whole egg model, the essential amino acid index (EAAI)of FMLP was as high as 95.67 ± 0.26%, meaning the essential amino acid composition was as close to that of whole eggs.

### 2.6. Changes in Volatile Compounds

**Figure 5:**
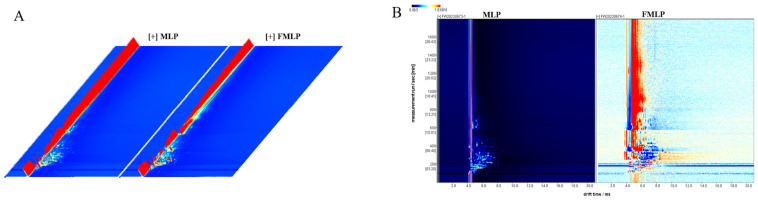
Spectrogram of GC-IMS. (**A**) Three-dimensional topographic plot. (**B**) Two-dimensional difference plot. The background is blue, and the red vertical line is the normalized reaction ion peak.

**Figure 6:**
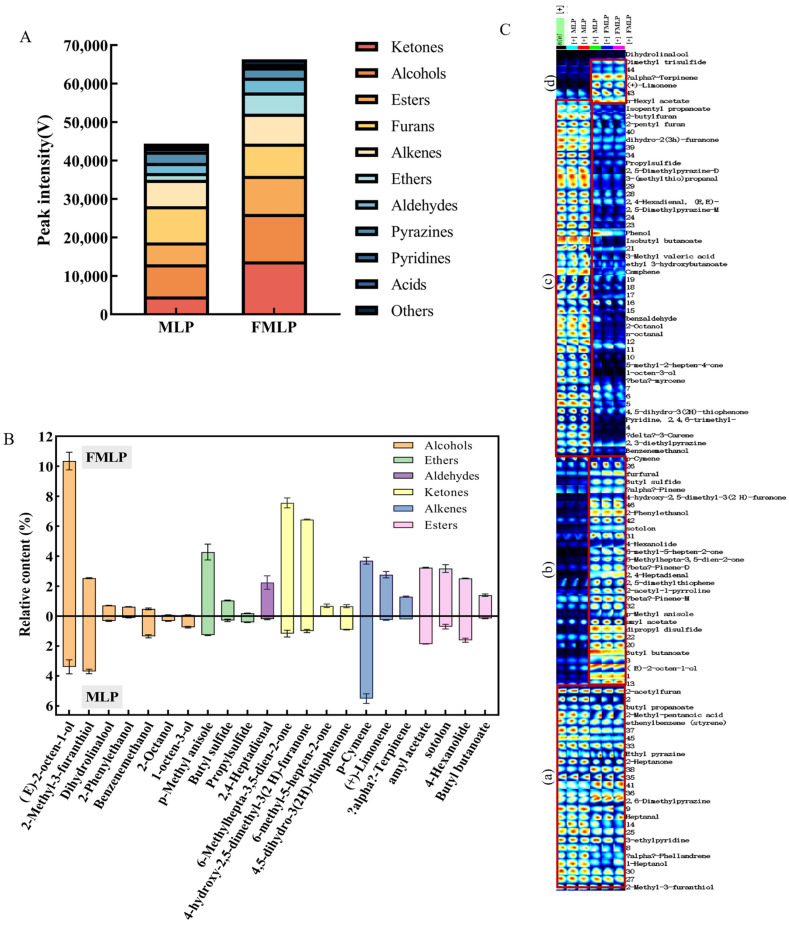
Volatile compounds observed by GC-IMS. (**A**) Peak intensities of total volatile compounds. (**B**) Changes in the content of volatile compounds before and after fermentation. (**C**) Gallery plots indicating the variations in VOCs’ relative content among the two groups. The color represents the concentration of the substance. Blue and red colors underline over- and under- expressed components.

GC-IMS is an effective method for the separation and detection of volatile compounds. Therefore, it was utilized in this experiment to compare the volatilizable substance composition of MLP and FMLP. Figure 5 shows the difference between the two mulberry leaf powder samples. A total of 62 compounds were identified by GC×IMS database search (Figure 6), including 8 alcohols, 7 aldehydes, 6 ketones, 10 esters, 2 acids, 3 furans, 10 alkenes, 5 pyrazines, 2 pyridines, 5 ethers, and 4 other compounds. The variations in the contents of these 62 flavor substances resulted in differences in flavor between MLP and FMLP (the relevant participant information is shown in Supplementary Table S2).

Alcohols are important substances constituting the characteristic flavor of mulberry leaves. Compared with MLP, the total amount of alcohols in FMLP increased by 47.83%. Among them, (E)-2-octen-1-ol, which has a citrus aroma, increased significantly from 3.39% to 10.35% after fermentation by *P. pentosaceus* JC30. In contrast, 1-octen-3-ol and 2-octanol presented earthy and grassy green flavors [46], and their contents decreased from 0.74% and 0.38% to 0.07% and 0.08%, respectively. As shown in the study, *P. pentosaceus* JC30 fermentation produced a citrus aroma that reduced the grassy and earthy flavors of the mulberry leaves.

The ketones were partly derived from the further oxidation of aldehydes. Compared with MLP, the total amount of ketones in FMLP increased by 195.6%. Among them, 6-methyl-3,5-dien-2-one with a sweet coconut aroma and flavor and 6-methyl-5-hepten-2-one with a fruity citrus aroma increased their relative contents by 5.45-fold and 33-fold, respectively. On the contrary, the content of 4,5-dihydro-3(2H)-thiophenone, which has a pungent garlic odor, decreased by 27.47%. The increase in ketone content in FMLP resulted in fruity and sweet caramel flavors.

Esters constitute the fruity flavor of mulberry leaves. The relative content of esters was 6.92% and 12.02% in MLP and FMLP, respectively. As shown in Figure 6C, compared with MLP, the contents of butyl butanoate and amyl acetate in FMLP increased by 8.33-fold and 0.73-fold, respectively, which were volatile at room temperature and had a greater potential to contribute to the flavor [47]. Therefore, the FMLP had a strong fruity odor.

Compared with MLP, FMLP contained 48.11% more aldehydes, 87.54% more alkenes, and 179.70% more ethers. The content of 2,4-heptadienal, which has a fruity odor, increased more than ten-fold. (+)-Limonene and α-terpinene are compounds with a fruity citrus odor, and their contents account for 70.12% of the total alkenes in FMLP, which was increased by 745.83% compared with MLP. Among the ether compounds, p-methyl anisole and butyl sulfide present floral and rose fragrance odors, and their contents in FMLP increased by 237.00% and 258.62%, respectively. Meanwhile, the content of propylsulfide, which has an irritating odor of garlic onion, decreased by 53.6%.

The contents of alcohols, esters, ketones, and aldehydes in FMLP significantly increased, thereby imparting a fruity and floral aroma while simultaneously attenuating the grassy and earthy flavor of the mulberry leaves. The findings indicated that *P. pentosaceus* JC30 demonstrated remarkable potential in improving the flavor of mulberry leaves. Zhuo et al. [48] reported that *P. pentosaceus* JS35 fermented mulberry leaf powder can produce floral and fruity odors. Xu et al. showed that the fermentation of *P. pentosaceus* changed the key odor substances and non-volatile metabolites of cauliflower juice and improved the sensory characteristics of cauliflower juice. So, *P. pentosaceus* JC30 is another strain with potential for flavor improvement.

## 3. Materials and Methods

### 3.1. Chemicals and Materials

Mulberry leaf powder (120 mesh) was purchased from Bozhou Hongyang Pharmaceutical Sales Co., Ltd. (Bozhou, China). *P. pentosaceus* JC30 was originally isolated from traditional kimchi coming from Changde, Hunan Province, China.

γ-Aminobutyric acid standard (≥99%) was purchased from Sigma Aldrich (St Louis, MO, USA). 1,1-Diphenyl-2-picrylhydrazyl free radical (DPPH) was purchased from TCI Chemical Industry Development Co., Ltd. (Shanghai, China). De Man, Rogosa, and Sharpe (MRS) broth and agar medium were purchased from Baisi Biotechnology (Hangzhou, China). Standard organic acids, including acetic acid, lactic acid, citric acid, succinic acid, oxalic acid, tartaric acid, malic acid, pyruvic acid, and pyroglutamic acid were bought from Shanghai Yuanye Bio-Technology Co., Ltd. (Shanghai, China). Standard substances of phenolic compounds, such as quercetin, hesperidin, arbutin, gallic acid, etc., were bought from Shanghai Yuanye Bio-Technology Co., Ltd. (Shanghai, China). Other chemical reagents were analytical grade and purchased from Sinopharm Chemical Reagent Co., Ltd. (Shanghai, China).

### 3.2. SSF of Mulberry Leaf Powder

The SSF nutrient medium was composed of 20 g of mulberry leaf powder, 80 mL of MRS medium, and 2 g of L-glutamic sodium. Before use, the medium was steam-sterilized in an autoclave at 121 °C for 20 min. *P. pentosaceus* JC30 was reactivated twice by culturing it at 37 °C for 18 h each time in MRS broth. The bacterial solution was collected and adjusted to a certain concentration (around 10^8^ CFU/mL), and 5% (*v*/*w*) *P. pentosaceus* JC30 was added to the SSF nutrient medium, which was mixed well with a sterile glass rod evenly and then placed in a constant temperature incubator at 37 °C for sealed fermentation for 48 h (fermented mulberry leaf powder, FMLP). The same mulberry leaf system as described above using distilled water to replace the bacterial solution was taken as the control (non-fermented mulberry leaf powder, MLP). All the samples were freeze-dried and stored for later use. Three replicates were used for all the experiments.

### 3.3. Determination of pH and Total Titratable Acidity

SSF nutrient medium was fermented at 37 °C by *P. pentosaceus* JC30. The pH value and total titratable acidity were determined every 12 h with a pH meter. The TTA was determined according to GB12456-2021 [49].

### 3.4. Determination of Organic Acids

According to the method of Jiang et al. [50], with slight modifications, 1 g of sample was added to 9 mL of 26 mmol/L sodium dihydrogen phosphate, sonicated for 5 min, and centrifuged to obtain the supernatant (8000 rpm, 10 min, 4 °C). The samples and standard working solution were filtered through a 0.22 μm filter membrane before use. Organic acid content was determined using an HPLC system (Agilent1260, Agilent Technologies, Santa Clara, CA, USA) equipped with a ZORBAX Eclipse Plus-C_18_ column (4.6 mm × 250 mm; 5 μm). The elution conditions were as follows. The elution was conducted with a mobile phase of 26 mmol/L sodium dihydrogen phosphate solution for 12 min. The column temperature was 30 °C, the detection wavelength was 210 nm, and the injection volume was 10 μL. Organic acids were identified and quantified by external standard methods. All determinations were repeated three times.

### 3.5. Determination of GABA, Total Sugar, Total Phenolic, and Total Flavonoid Contents

The water extract of the sample was prepared according to the method of Jin et al. [51], with slight modifications. Freeze-dried powder (1 g) was immersed in 40 mL of distilled water (100 °C) and extracted for 40 min at 70 °C and then centrifuged at 8000 rpm for 10 min to collect the supernatants. The extraction was repeated once using the same procedure. The two supernatants were then combined to obtain the water extract. The method of GABA determination was performed according to Jin et al. [51], and 0.0~0.5 mg/mL GABA was selected for preparing standard curve (y = 1.8852x − 0.0185, R^2^ = 0.9987).

Freeze-dried powder (1 g) was immersed in 40 mL of distilled water (100 °C) and extracted for 40 h at 60 °C and then centrifuged at 8000 rpm for 10 min to collect the supernatants. The extraction was repeated once using the same procedure. The two supernatants were then combined to obtain the water extract. The total sugar content was determined by phenol/sulfuric acid method. Specifically, 1 mL of 50 mg/mL phenol solution and 5 mL of concentrated sulfuric acid were successively added to 2 mL of the sample and reacted for 30 min at 40 °C. The sample was taken out, cooled to room temperature, and the absorbance was measured at 490 nm. Total sugar was expressed as glucose of samples through glucose calibration curve calibration (0.0~0.2 mg/mL, y = 5.2577x − 0.0056, R^2^ = 0.9986).

The ethanol extract of the sample was prepared according to the method of Eom et al. [52], with slight modification. Freeze-dried powder (1 g) was added to 40 mL of ethanol solution (70%) and then extracted for 1 h at 60 °C. The supernatant was collected by centrifugation at 8000 rpm for 10 min. The extraction was repeated once following the same procedure. Subsequently, the two supernatants were combined to obtain an ethanol extract for determination of total phenolic and total flavonoid contents.

TPC was determined by the Folin–Ciocalteu method according to Tu et al. [53]. 100 μL of the extract was added with 100 μL of Folin–Ciocalteu reagent and 200 μL of 10% Na_2_CO_3_ solution and diluted to 10 mL with distilled water. A 40 °C water bath was used for 1 h. The absorbance at 760 nm was measured. TPC was expressed as the gallic acid equivalents (GAE) of the samples through gallic acid standard curve calibration (0.0–0.10 mg/mL, y = 9.5573x − 0.1281, R^2^ = 0.9980). TFC was measured using the aluminum chloride colorimetric method with absorbance at 510 nm, as described by Zhuo et al. [48]. Then, 200 μL of extract was added to 30 μL of 5% NaNO_2_, shaken well, and allowed to stand for 6 min, and 30 μL of 10% AlCI_3_ solution was added, shaken well, and allowed to stand for 6 min. Then, 200 μL of 40 mg/mL NaOH solution was added. After shaking well, the solution was diluted with 40% ethanol solution to 1 mL, and the absorbance at 510 nm was measured after 10 min of standing. TFC was expressed as the rutin equivalent (mg RE/100g) of the samples through rutin calibration curve calibration (0.0~0.6 mg/mL, y = 1.2953x + 0.0137, R^2^ = 0.9993).

### 3.6. Determination of Phytic Acid Contents

The determination of phytic acid was according to the method of Kahriman et al. with slightly modified [54]. Mulberry leaf powder (1 g) was shaken in 20 mL of 39.67 mg/mL HCl and 100 mg/mL Na_2_SO_4_ for 2 h at room temperature and then centrifuged at 5000 rpm for 10 min. The supernatant was obtained and the volume was adjusted to 50 mL.

A total of 2 mL of extract was added to 2 mL of 150 mg/mL trichloroacetic acid and stood at 4 °C for 2 h after shaking. The extract mixture was subsequently centrifuged at 4000× *g* for 10 min, after which 2 mL of the supernatant was adjusted to pH 6.0–6.5 with 30 mg/mL NaOH and diluted to 30 mL. Then, 3 mL of the diluted solution was mixed with 1 mL of the reaction mixture (the reaction mixture was a mixture of 3 mg/mL sulfosalicylic acid and 0.3 mg/mL FeCl_3_•6H_2_O in equal proportions). The absorbance at 500 nm was determined. Through the calibration curve of sodium phytate (0.0~0.1 mg/mL), the phytic acid content was expressed as sodium phytate (mg/g) in the samples (y = −2.1319x + 0.4079, R^2^ = 0.9990).

### 3.7. Analysis of Phenolic Compounds of Mulberry Leaf Powder

#### 3.7.1. Extraction of Free Phenolic Compounds from Mulberry Leaf Powder

The phenolic compounds of the samples were prepared according to the method of Wang et al. [55], with slight modifications. A 2.0 g sample was added to 50 mL of precooled methanol (80%). The mixture was extracted by homogenization (5000 rpm, 10 min) and centrifugation (7000 rpm, 5 min). The above extraction was repeated 3 times, and the supernatants were mixed. Then, the collected supernatants were evaporated to dryness at 45 °C using a rotary evaporator. The dried extract was dissolved with water to a constant volume of 10 mL, filtered through a 0.22 μm membrane filter, and stored at −20 °C until use. Mulberry leaf powder precipitates were collected for the further extraction of bound phenolic compounds.

#### 3.7.2. Extraction of Bound Phenolic Compounds from Mulberry Leaf Powder

The above residue of mulberry leaf powder was digested with 2 M NaOH for 1.5 h at room temperature and shaken under nitrogen. The pH was adjusted to 2.0 with HCl (6 M) and the mixture was extracted with n-hexane to remove lipids. The final solution was extracted with ethyl acetate 6 times. The combined ethyl acetate extract was collected as bound phenolic compounds. The extracts were evaporated to dryness at 45 °C, and the dried extract was dissolved in water to a constant volume of 10 mL. All the dissolved extracts were filtered through a 0.22 μm membrane filter and stored at −20 °C until use.

#### 3.7.3. UPLC-Q-TOF-MS Analysis

All samples were filtered through 0.22 μm filter membrane and determined on a UPLC-Q-TOF-MS system (Waters Q-TOF Synapt G2 high-resolution mass spectrometer and Waters Acquity UPLC system, Water, Milford, MA, USA). An Acquity UHPLC BEH C_18_ column (5 μm, 2.1 × 150 mm) equipped with an electrospray ionization (ESl) unit was used for phenolic compound analysis. The liquid chromatography conditions were as follows: column temperature 35 °C and injection volume 5 μL. Solvent A consisted of water with 0.1% formic acid, and solvent B was acetonitrile. Chromatography was carried out at a flow rate of 0.3 mL/min. A linear gradient was programmed for 30 min as follows: 0–22min, 99–30% A; 22–24 min, 30–100% A; 24–26 min, 100%A; 26–27 min, 100–99% A; 27–30 min, 100% A. The mass spectrometry conditions were as follows: the electrospray ion source has capillary voltages of 3.0 kV and 2.0 kV for positive (ESI^+^) and negative (ESI^−^) ions, respectively, with a mass range of 50–1000 and a resolution of 5000. The desolvation gas was (N_2_) 800 L/h at 550 °C; the cone gas was 50 L/h; and the ion source temperature was 150 °C. Collision energy for obtaining MS/MS spectra was set between 10 and 30 V, using leucine enkephalin (Leu-Enk, *m*/*z* 554.2620, [M-H]^−^) for calibration.

### 3.8. Evaluation of Antioxidant Activities

#### 3.8.1. DPPH Radical Scavenging Assay

The DPPH radical scavenging capacity of fermented and unfermented mulberry leaf powder was assayed using the method described by Jin et al. [51]. Briefly, 1 mL of sample and 1 mL of DPPH/methanol solution were mixed homogeneously, and the absorbance value was measured at 517 nm after 30 min of reaction at room temperature and protected from light, as the sample group (A_517_); 1 mL of DPPH solution added with 1 mL of methanol was used as the control group (A_max_); 1 mL of the same concentration of sample and 1 mL of methanol were used as the blank group (A_0_); and vitamin C was used as the positive control. The calculation formula is as follows:

$$\mathrm{DPPH}\;\mathrm{radical}\;\mathrm{scavenging}\;\mathrm{cappacity}\left(\%\right)=\left(1-\frac{A_{517}-A_{0}}{A_{\max}}\right)\times 100$$

#### 3.8.2. Hydroxyl Radical Scavenging Assay

The hydroxyl radical scavenging capacity was assayed using the method described by Zhuo et al. [48]. Briefly, 1 mL of 9 mmol/L ferrous sulfate solution, 1 mL of 9 mmol/L salicylic acid ethanol solution, 1mL of sample solutions with different concentrations, and 1 mL of 0.1% hydrogen peroxide solution were added in turn. After incubation at 37 °C for 30 min, the solutions were centrifuged at 7000 r/min for 3 min. The absorbance was measured at 510 nm(A_510_). The control group (A_max_) was distilled water instead of sample solution. The blank group (A_0_) was distilled water instead of 0.1% (*v*/*v*) hydrogen peroxide solution. Vitamin C was used as a positive control. The calculation formula is as follows:

$$\mathrm{Hydroxyl}\;\mathrm{radical}\;\mathrm{scavenging}\;\mathrm{assay}\left(\%\right)=\left(1-\frac{A_{510}-A_{0}}{A_{\max}}\right)\times 100$$

#### 3.8.3. Total Reducing Power Assay

The total reducing power was determined from the method of Xiao et al. [56]. with some modifications. Briefly, 1 mL of sample extract was mixed with 1 mL of phosphate buffer (0.2 M, pH 6.6) and 1 mL of potassium ferricyanide solution (10 mg/mL), and then the mixture was heated at 50 °C for 20 min. Subsequently, 1 mL of trichloroacetic acid (100 mg/mL) was added, and the sample was subjected to centrifugation at 3000 rpm for 10 min. Then, 1 mL of the supernatant was mixed with 0.2 mL of FeCl_3_ (1 mg/mL) and 1 mL of distilled water and then incubated for 10 min. The absorbance was measured at 700 nm. Vitamin C was used as a positive control.

### 3.9. Determination of FAAs and Evaluation of Nutritional Value

#### 3.9.1. Determination of FAAs

The sample extraction and determination methods referred to GB/T 30987-2020 [57]. The supernatant was analyzed with a Hitachi L-8900 automatic amino acid analyzer (Hitachi Ltd., Tokyo, Japan). The analytical conditions were as follows: analytical column, 2622PH 4.6 mm I.D. × 60 mm; flow rate, 0.30 mL/min; reaction temperature, 135 °C; detection wavelengths, 570 nm and 440 nm; injection volume, 20 µL.

#### 3.9.2. Evaluation of Nutritional Value of Amino Acids

Nutritional evaluation was performed according to the ideal protein human essential amino acid model recommended by the Food and Agriculture Organization of the United Nations (FAO) and the World Health Organization (WHO) in 1973 and the egg protein model proposed by the Institute of Nutrition and Food Hygiene, Chinese Academy of Preventive Medicine. Amino acid score (AAS), chemical score (CS), and essential amino acid index (EAAI) were calculated according to the formula [44,58].

$$\mathrm{AAS}=\frac{\mathrm{The}\;\mathrm{content}\;\mathrm{of}\;\mathrm{an}\;\mathrm{amino}\;\mathrm{acid}\;\mathrm{in}\;\mathrm{the}\;\mathrm{protein}\;\mathrm{to}\;\mathrm{be}\;\mathrm{tested}}{\mathrm{FAO}/\mathrm{WHO}\;\mathrm{scoring}\;\mathrm{standard}\;\mathrm{model}\;\mathrm{of}\;\mathrm{the}\;\mathrm{same}\;\mathrm{amino}\;\mathrm{acid}\;\mathrm{content}}\times 100$$

$$\mathrm{CS}=\frac{\mathrm{The}\;\mathrm{content}\;\mathrm{of}\;\mathrm{an}\;\mathrm{amino}\;\mathrm{acid}\;\mathrm{in}\;\mathrm{the}\;\mathrm{protein}\;\mathrm{to}\;\mathrm{be}\;\mathrm{tested}}{\mathrm{The}\;\mathrm{same}\;\mathrm{amino}\;\mathrm{acid}\;\mathrm{content}\;\mathrm{in}\;\mathrm{egg}\;\mathrm{protein}}\times 100$$

$$\mathrm{EAAI}=\sqrt[{n}]{\frac{{\mathrm{Lysine}}_{pro}\times {\mathrm{leucine}}_{pro}\times \cdots \times {\mathrm{valine}}_{pro}\times {\mathrm{trptophan}}_{pro}}{{\mathrm{Lysine}}_{\mathrm{ep}}\times {\mathrm{leucine}}_{\mathrm{ep}}\times \cdots \times {\mathrm{valine}}_{ep}\times {\mathrm{trptophan}}_{\mathrm{ep}}}}\times 100$$

where n is the number of amino acids compared, “pro” is the amino acid content of the protein to be evaluated, “ep” is the amino acid content of egg protein.

### 3.10. Flavor Analysis of Mulberry Leaf Powder

The GC-IMS analysis was conducted on the FlavourSpec^®^ GC-IMS device (G.A.S. Dortmund, Germany). Briefly, 2 g of sample was added to a 20 mL headspace vial and incubated at 60 °C for 20 min. After incubation, 500 μL of the headspace sample was automatically injected into the syringe (no shunt mode) through a heated syringe at 85 °C. The GC was performed with MXT-5 capillary column (15 m × 0.53 mm; column temperature: 60 °C) to separate volatile components and coupled to IMS at 45 °C. Gas chromatography conditions: nitrogen as carrier gas, 0–10 min flow rate of 2 mL/min, 10–20 min flow rate of 10 mL/min, 20–30 min flow rate of 100 mL/min; the final flow rate increased to 150 mL/min.

The built-in NIST 2014 gas-phase retention index database and G.A.S IMS migration time database were used for qualitative analysis. The relative content of each component was processed by peak area normalization.

### 3.11. Statistical Analysis

The results were presented as mean ± standard deviation (SD) from at least triplicate analyses. The data were analyzed and plotted using GraphPad Prism 8.0 and Excel. A one-way ANOVA was performed with SPSS 26.0 (SPSS, Chicago, IL, USA). Duncan’s method was used to test the significance of differences in the data (*p* < 0.05, significant difference).

## 4. Conclusions

Fermentation by *P. pentosaceus* JC30 is an effective way to improve phytochemical, flavor characteristics and antioxidant activity of mulberry leaves. *P. pentosaceus* JC30 fermentation increased the acidity and the contents of organic acids in the mulberry leaf powder, especially lactic acid and citric acid, which increased by 15.79- and 11.99-fold, respectively. The fermentation increased the contents of GABA and total phenolics, the content of GABA in FMLP was increased 6.73-fold, and the total free phenolics was increased 88.43%, especially that of leucocyanidin, ellagic acid_1, myricetin, and quercetin in the free state, which increased by 117.25-, 39.80-, 27.95-, and 11.70-fold, respectively. The content of the anti-nutritional factor phytic acid in mulberry leaves was decreased by 11.16%. The fermentation also increased the content of EAA; the contents of Met, Leu, and Lys were increased 18.73-, 7.41-, and 3.49-fold, respectively. The AAS, CS, and EAAI values of FMLP were all higher than those of MLP. Additionally, the fermentation improved the flavor of mulberry leaf powder, with a significant increase in (E)-2-octen-1-ol, (+)-limonene, p-methyl anisole, etc., which have citrus and floral aromas, while reducing the grassy, green, and earthy flavors. The fermentation increased the antioxidant capacity of mulberry leaf powder; the DPPH radical scavenging capacity of FMLP reached 80.30 ± 1.54% at a concentration of 0.5 mg/mL. In summary, *P. pentosaceus* JC30 fermentation improved the nutritional value, flavor, and antioxidant activity of mulberry leaves, which has potential for application to the development of functional foods. The fermented mulberry leaves can be used as dietary supplements or high-value-added feed products. Subsequent studies can further evaluate the bioavailability and health efficacy of FMLP in animal models or human trials, providing a scientific basis for its wide application in the field of food and feed.

## Figures and Tables

**Table 1:** Organic acid contents of the MLP and FMLP samples (mg/g).

Organic Acids	MLP	FMLP
Lactic acid	ND	15.79 ± 0.40 ^a^
Acetic acid	0.68 ± 0.04 ^b^	4.02 ± 0.22 ^a^
Citric acid	0.19 ± 0.00 ^b^	2.44 ± 0.09 ^a^
Malic acid	2.86± 0.31 ^a^	0.13 ± 0.05 ^b^
Tartaric acid	0.11 ± 0.00 ^b^	0.98 ± 0.12 ^a^
Pyruvic acid	0.01 ± 0.00 ^b^	0.22 ± 0.10 ^a^
Oxalic acid	5.38 ± 0.17 ^a^	5.60 ± 0.33 ^a^
Pyroglutamic acid	3.33 ± 0.23 ^a^	0.23 ± 0.03 ^b^
Succinic acid	3.88 ± 0.44 ^a^	0.79 ± 0.13 ^b^

ND, not detected. Values in the same row with different superscripted letters are significantly different at *p* < 0.05.

**Table 2:** Changes in free phenolics and bound phenolics in mulberry leaves before and after fermentation.

Name	Relative Content (%)			
	BPs (MLP)	FPs (MLP)	BPs (FMLP)	FPs (FMLP)
3,4-Dihydroxycinnamic acid	2.10 ± 0.57	2.17 ± 0.16	0.53 ± 0.03	2.88 ± 0.12
Arundinin	0.15 ± 0.01	0.31 ± 0.04	ND	0.09 ± 0.00
Chlorogenic acid	ND	0.22 ± 0.02	ND	0.19 ± 0.02
Dihydrocoumarin	0.02 ± 0.00	0.76 ± 0.05	0.01 ± 0.00	0.32 ± 0.02
Ellagic acid_1	0.07 ± 0.01	0.09 ± 0.01	ND	3.76 ± 0.00
Gallic acid	0.23 ± 0.03	0.47 ± 0.02	0.10 ± 0.02	0.21 ± 0.01
Hesperidin	0.06 ± 0.01	0.29 ± 0.01	0.08 ± 0.02	0.03 ± 0.00
Leucocyanidin	1.69 ± 0.02	0.03 ± 0.01	0.37 ± 0.01	3.71 ± 0.01
Myricetin	0.26 ± 0.02	0.36 ± 0.03	0.01 ± 0.00	10.44 ± 0.02
Naringenin	0.05 ± 0.02	0.62 ± 0.02	ND	0.03 ± 0.01
Neohesperidin	6.70 ± 0.03	3.10 ± 0.82	ND	5.65 ± 0.01
Procyanidin B_6_	ND	0.07 ± 0.01	ND	0.30 ± 0.01
Protocatechuic acid	16.03 ± 0.01	0.11 ± 0.00	0.14 ± 0.01	0.81 ± 0.01
Quercetin	12.60 ± 0.03	3.19 ± 0.03	ND	40.55 ± 0.01
Rosavin	0.29 ± 0.01	0.05 ± 0.01	0.02 ± 0.00	0.51 ± 0.01

ND, not detected. BPs, bound phenolics; FPs, free phenolics.

**Table 3:** Composition and content of free amino acids in MLP and FMLP.

Parameter (mg/100 g)	MLP	FMLP
Lysine (Lys)	17.32 ± 0.07 ^b^	77.76 ± 0.08 ^a^
Phenylalanine (Phe)	12.90 ± 0.16 ^b^	58.00 ± 0.61 ^a^
Methionine (Met)	ND	18.73 ± 0.10 ^a^
Threonine (Thr)	36.58 ± 0.93 ^b^	51.23 ± 0.28 ^a^
Leucine (Leu)	9.97 ± 0.10 ^b^	83.81 ± 0.18 ^a^
Isoleucine (Ile)	10.55 ± 0.11 ^b^	44.60 ± 0.07 ^a^
Valine (Val)	29.79 ± 0.03 ^b^	90.90 ± 0.10 ^a^
Histidine (His)	12.48 ± 0.05 ^b^	16.42 ± 0.83 ^a^
Arginine (Arg)	26.02 ± 0.40 ^a^	4.12 ± 0.00 ^b^
Glycine (Gly)	11.05 ± 0.01 ^b^	97.56 ± 0.12 ^a^
Alanine (Ala)	33.42 ± 0.15 ^b^	157.30 ± 0.45 ^a^
Proline (Pro)	119.63 ± 0.97 ^b^	106.94 ± 0.80 ^a^
Tyrosine (Tyr)	ND	22.94 ± 0.50 ^a^
Serine (Ser)	11.21 ± 0.02 ^b^	17.52 ± 0.18 ^a^
Aspartic acid (Asp)	54.59 ± 0.04 ^b^	143.84 ± 0.13 ^a^
Cystine (Cys)	21.87 ± 0.06 ^a^	22.44 ± 0.01 ^a^
TAAs	407.38 ± 3.10 ^b^	1014.61 ± 4.44 ^a^
EAAs	117.11 ± 1.40 ^b^	425.03 ± 1.42 ^a^
NEAAs	290.27 ±1.66 ^b^	589.58 ± 3.00 ^a^
EAAs/TAAs (%)	28.75 ± 0.35 ^b^	41.89 ± 0.14 ^a^
EAAs/NEAAs (%)	40.35 ± 0.48 ^b^	72.09 ± 0.24 ^a^

TAAs: total amino acids; EAAs: essential amino acids; NEAAs: non-essential amino acids. ND: not detected. Values in the same row with different superscripted letters are significantly different at *p* < 0.05.

**Table 4:** Evaluation of amino acid nutritional value of MLP and FMLP.

	FAO/WHO Recommended Pattern (%)	Whole Egg Protein Pattern (%)	% of Total Amino Acids		AAS (%)		CS (%)	
			MLP	FMLP	MLP	FMLP	MLP	FMLP
Ile	4.00	5.30	2.59 ± 0.03	4.40 ± 0.01	64.74 ± 0.68	109.89 ± 0.17	48.86 ± 0.51	82.94 ± 0.13
Leu	7.00	8.60	2.45 ± 0.02	8.26 ± 0.02	34.96 ± 0.35	118.00 ± 0.25	28.46 ± 0.29	96.05 ± 0.21
Lys	5.50	7.00	4.25 ± 0.02	7.66 ± 0.01	77.30 ± 0.31	139.35 ± 0.14	60.74 ± 0.25	109.49 ± 0.11
Met+Cys	3.50	6.17	5.37 ± 0.01	4.06 ± 0.01	153.38 ± 0.42	115.93 ± 0.31	87.01 ± 0.24	65.77 ± 0.18
Phe+Tyr	6.00	9.04	3.17 ± 0.13	7.93 ± 0.06	52.78 ± 0.65	132.11 ± 0.92	35.03 ± 0.43	87.68 ± 0.61
Thr	4.00	4.70	8.98 ± 0.13	5.05 ± 0.03	224.48 ± 3.21	126.23 ± 0.69	191.05 ± 2.73	107.43 ± 0.59
Val	5.00	6.60	7.31 ± 0.01	8.96 ± 0.01	146.25 ± 0.15	179.18 ± 0.20	110.80 ± 0.11	135.74 ± 0.15
EAAI (%)	/	/	65.99 ± 0.19	95.67 ± 0.26	/	/	/	/

## Data Availability

Data will be made available on request.

## Supplementary Materials

The following supporting information can be downloaded at: https://www.mdpi.com/article/10.3390/molecules30081703/s1, Table S1: The composition and relative content of free phenolics and bound phenolics in mulberry leaves were identified by UPLC-Q-TOF-MS; Table S2: Volatile compounds identified in mulberry leaf power by GC-IMS.

## Abbreviations

The following abbreviations are used in this manuscript:

GABA	γ-aminobutyric acid
LAB	Lactic acid bacteria
P. pentosaceus	Pediococcus pentosaceus
SSF	Solid-state fermentation
FMLP	Fermented mulberry leaf powder
MLP	Non-fermented mulberry leaf powder
TTA	Total titratable acidity
TPC	Total phenolic content
TFC	Total flavonoid content
DPPH	1,1-Diphenyl-2-picrylhydrazyl free radical
•OH	Hydroxyl radical
TRP	Total reducing power
BPs	Bound phenolics
FPs	Free phenolics
FAAs	Free amino acids
AAS	Amino acid score
CS	Chemical score
EAAI	Essential amino acid index
TAAs	Total amino acids
EAAs	Essential l amino acids
NEAAs	Non-essential amino acids
