# Supplementary material for: Fermentation of Pediococcus pentosaceus JC30 Improves Phytochemical, Flavor Characteristics and Antioxidant Activity of Mulberry Leaves
Source: Molecules. 2025 Apr 10;30(8):1703. doi: 10.3390/molecules30081703 (PMC12029543; doi:10.3390/molecules30081703)
Supplement: Supplementary file 1 [file molecules-30-01703-s001.zip › molecules-3521979-supplementary/supplementary/Supplementary material Table S2.pdf]

Table S2

Volatile compounds identified in mulberry leaf power by GC-IMS

Compound	CAS#	Formula	RI	Rt [sec]	Dt [a.u.]	Relative content (%)		VIP
						MLP	FMLP	
**Ketones**								
2-Heptanone	C110430	$C_7H_{14}O$	903.4	159.773	1.248	2.02±0.04	1.37±0.03	0.90
4,5-dihydro-3(2H)-thiophenone	C1003049	C_4H_6OS	982.2	303.707	1.182	0.91±0.01	0.66±0.10	0.53
4-hydroxy-2,5-dimethyl-3(2 H)-furanone	C3658773	$C_6H_8O_3$	1064.8	594.792	1.203	1.00±0.11	6.44±0.03	2.60
5-methyl-2-hepten-4-one	C81925817	$C_8H_{14}O$	969.7	274.191	1.691	0.56±0.08	0.09±0.04	0.75
6-methyl-5-hepten-2-one	C110930	$C_8H_{14}O$	996.8	341.884	1.166	0.02±0.00	0.68±0.12	0.89
6-Methylhepta-3,5-dien-2-one	C1604280	$C_8H_{12}O$	1102.5	808.794	1.212	1.17±0.22	7.55±0.33	2.81
**Alcohols**								
(E)-2-octen-1-ol	C18409171	$C_8H_{16}O$	1068.6	613.399	1.154	3.39±1.44	10.35±0.59	2.89
1-Heptanol	C111706	$C_7H_{16}O$	969.4	273.62	1.393	0.24±0.03	0.18±0.03	0.24
1-octen-3-ol	C3391864	$C_8H_{16}O$	974.8	285.793	1.602	0.74±0.05	0.07±0.01	0.91
2-Methyl-3-furanthiol	C28588741	C_5H_6OS	876.9	130.57	1.144	3.70±0.15	2.53±0.03	1.19
2-Octanol	C123966	$C_8H_{18}O$	988.3	319.126	1.440	0.32±0.02	0.08±0.01	0.55
2-Phenylethanol	C60128	$C_8H_{10}O$	1101.7	803.607	1.291	0.11±0.01	0.62±0.01	0.80
Benzenemethanol	C100516	C_7H_8O	1025.7	432.54	1.330	1.35±0.11	0.48±0.06	1.04

Table S2 (continued)

Compound	CAS#	Formula	RI	Rt [sec]	Dt [a.u.]	Relative content (%)		VIP
						MLP	FMLP	
Dihydrolinalool	C78693	$C_{10}H_{22}O$	1123.5	959.642	1.269	0.31±0.04	0.71±0.01	0.70
**Esters**								
4-Hexanolide	C695067	$C_6H_{10}O_2$	1074.2	642.038	1.194	1.61±0.14	2.51±0.02	1.05
Amyl acetate	C628637	$C_7H_{14}O_2$	927.2	193.974	1.314	1.87±0.00	3.23±0.04	1.30
Butyl butanoate	C109217	$C_8H_{16}O_2$	1000	351.056	1.333	0.15±0.04	1.40±0.08	1.24
Butyl propanoate	C590012	$C_7H_{14}O_2$	901.4	157.243	1.292	0.54±0.02	0.60±0.00	0.27
Dihydro-2(3h)-furanone	C96480	$C_4H_6O_2$	913.2	173.071	1.084	0.37±0.06	0.23±0.01	0.39
Ethyl 3-hydroxybutanoate	C5405414	$C_6H_{12}O_3$	946.4	226.932	1.172	0.29±0.02	0.17±0.00	0.37
Isobutyl butanoate	C539902	$C_8H_{16}O_2$	961.8	257.234	1.347	0.49±0.00	0.12±0.06	0.67
Isopentyl propanoate	C105680	$C_8H_{16}O_2$	974.5	285.06	1.353	0.67±0.04	0.28±0.09	0.69
n-Hexyl acetate	C142927	$C_8H_{16}O_2$	1013.4	391.425	1.380	0.22±0.02	0.31±0.02	0.33
Sotolon	C28664359	$C_6H_8O_3$	1118.3	919.445	1.218	0.71±0.15	3.17±0.25	1.74
**Alkenes**								
(+)-Limonene	C138863	$C_{10}H_{16}$	1006.8	370.862	1.282	0.26±0.02	2.76±0.21	1.76
α-Phellandrene	C99832	$C_{10}H_{16}$	1036.1	470.823	1.230	0.60±0.07	0.38±0.01	0.50
α-Pinene	C80568	$C_{10}H_{16}$	920.9	184.243	1.209	0.19±0.01	0.24±0.00	0.25
α-Terpinene	C99865	$C_{10}H_{16}$	1006.5	370.029	1.231	0.22±0.00	1.30±0.03	1.16
β-myrcene	C123353	$C_{10}H_{16}$	967.2	268.774	1.289	0.63±0.04	0.29±0.06	0.64
β-Pinene-D	C127913	$C_{10}H_{16}$	951.2	235.844	1.738	0.10±0.01	0.33±0.01	0.54

Table S2 (continued)

Compound	CAS#	Formula	RI	Rt [sec]	Dt [a.u.]	Relative content (%)		VIP
						MLP	FMLP	
β-Pinene-M	C127913	$C_{10}H_{16}$	951.2	235.797	1.298	0.16±0.01	0.25±0.01	0.33
β-3-Carene	C13466789	$C_{10}H_{16}$	1013.2	390.714	1.636	0.43±0.05	0.06±0.01	0.67
Camphene	C79925	$C_{10}H_{16}$	946	226.049	1.212	0.11±0.01	0.06±0.01	0.23
Ethenylbenzene (styrene)	C100425	C_8H_8	894.6	148.794	1.051	0.11±0.00	0.12±0.00	0.07
**Aldehydes**								
2,4-Heptadienal	C5910850	$C_7H_{10}O$	1001.2	354.435	1.187	0.20±0.04	2.24±0.45	1.57
2,4-Hexadienal, (E, E)-	C142836	C_6H_8O	924.4	189.632	1.115	0.62±0.02	0.45±0.01	0.45
3-(methylthio)propanal	C3268493	C_4H_8OS	918.6	180.922	1.392	0.36±0.01	0.22±0.00	0.42
Benzaldehyde	C100527	C_7H_6O	968.4	271.371	1.468	0.53±0.06	0.24±0.12	0.56
Furfural	C98011	$C_5H_4O_2$	857	113.102	1.092	0.40±0.02	0.69±0.08	0.58
Heptanal	C111717	$C_7H_{14}O$	905.4	162.389	1.335	0.71±0.02	0.75±0.01	0.23
n-octanal	C124130	$C_8H_{16}O$	1001.2	354.383	1.403	0.36±0.01	0.12±0.00	0.55
**Ethers**								
Butyl sulfide	C544401	$C_8H_{18}S$	1081.4	681.067	1.292	0.29±0.08	1.04±0.02	0.96
Dimethyl trisulfide	C3658808	$C_2H_6S_3$	980.3	298.918	1.301	0.15±0.01	0.87±0.14	0.94
Dipropyl disulfide	C629196	$C_6H_{14}S_2$	1102.2	806.663	1.261	0.06±0.01	0.33±0.04	0.57
p-Methyl anisole	C104938	$C_8H_{10}O$	1017.9	406.036	1.108	1.27±0.03	4.28±0.53	1.91
Propylsulfide	C111477	$C_6H_{14}S$	891.7	145.4	1.159	0.41±0.02	0.19±0.02	0.53
**Furans**								
2-acetylfuran	C1192627	$C_6H_6O_2$	888.5	142.005	1.118	9.94±0.11	9.42±0.10	0.78
2-butylfuran	C4466244	$C_8H_{12}O$	897.2	151.94	1.173	0.20±0.02	0.14±0.02	0.26
2-pentyl furan	C3777693	$C_9H_{14}O$	991.7	328.087	1.254	1.36±0.01	0.63±0.07	0.95
**Acids**								
2-Methyl-pentanoic acid	C97610	$C_6H_{12}O_2$	946.8	227.656	1.266	0.40±0.04	0.36±0.03	0.19
3-Methyl valeric acid	C105431	$C_6H_{12}O_2$	940.3	215.764	1.277	0.31±0.08	0.16±0.01	0.39
**Pyrazines**								
2,3-diethylpyrazine	C15707241	$C_8H_{12}N_2$	1085.6	704.374	1.225	1.01±0.16	0.67±0.01	0.62
2,5-Dimethylpyrazine-D	C123320	$C_6H_8N_2$	929.2	197.185	1.492	0.14±0.01	0.04±0.00	0.36
2,5-Dimethylpyrazine-M	C123320	$C_6H_8N_2$	931.3	200.65	1.119	0.29±0.02	0.09±0.00	0.50
2,6-Dimethylpyrazine	C108509	$C_6H_8N_2$	909.3	167.72	1.139	1.57±0.04	1.84±0.06	0.57
Ethyl pyrazine	C13925003	$C_6H_8N_2$	928.3	195.805	1.149	0.57±0.01	0.56±0.03	0.04
**Pyridines**								
3-ethylpyridine	C536787	C_7H_9N	954.4	242.057	1.109	0.39±0.01	0.40±0.08	0.04
Pyridine, 2,4,6-trimethyl-	C108758	$C_8H_{11}N$	986.5	314.484	1.150	0.82±0.05	0.19±0.02	0.88
**Others**								
2,5-dimethylthiophene	C638028	C_6H_8S	875.4	129.179	1.069	0.14±0.01	0.35±0.03	0.51
2-acetyl-1-pyrroline	C85213225	C_6H_9NO	916.9	178.428	1.482	0.29±0.03	1.01±0.05	0.94
p-Cymene	C99876	$C_{10}H_{14}$	1023.6	425.259	1.180	5.51±0.32	3.70±0.23	1.48
Phenol	C108952	C_6H_6O	967.1	268.395	1.075	0.18±0.02	0.13±0.02	0.23
